# The Physical Anomalies of Feebleminded Children

**Published:** 1906-02-10

**Authors:** 


### THE PHYSICAL ANOMALIES OF FEEBLE-MINDED CHILDREN.

Dr. C. Paget Lapage [1] has recently published the results of a careful statistical inquiry into the frequency with which certain physical anomalies appear in association with intellectual deficiency. The occurrence of these stigmata of degeneracy has long been recognised, but their existence throws an interesting sidelight on the widespread nature of the developmental disturbance which finds its most striking expression in that part of the economy where departure from the normal is taxed most heavily.

In a study based upon the observation of some hundreds of feeble-minded children in an institution for the reception of such victims, this author states that no fewer than 90 per cent. present some physical anomaly. But single anomalies are common outside the ranks of the feeble-minded; physical anomalies achieve intrinsic importance as indications of a generalised failure only when they occur in combinations of two or more. In the majority of cases there is some degree of microcephaly, this condition presenting itself in 34 per cent. of the series of cases. In association with microcephaly it is common to find some degree of asymmetry of the skull, the right half being generally larger than the left. In addition, it is quite frequent to find a well-marked deficiency of the posterior parts of the skull, although this condition is, of course, no rarity among people of average mental calibre.

In 32 per cent. of the cases there was some unusual appearance of the external ear, variations in the lobule being the most frequent departure from the common form. In 8 per cent. there were found "epicanthal folds," "everted ridges of skin continued round the internal angle of the eye from the upper eyelid."

Of all structures the palate is the most commonly deformed. It may be V-shaped, saddle-shaped, or highly arched. One or other of these defects was apparent in 67 per cent. of the cases, and of these varieties the saddle-shaped and the highly arched were the most common, these two together amounting to 57 per cent. Palatal defects are often to be observed in children of good mental calibre, and are in the author's opinion rather an indication of neuropathic inheritance than of anything more definite. Like the other defects here considered, these acquire a real significance as an indication of mental failure only when they occur in association with some one or more of the other common departures from the normal.

Obliquity of the orbits was observed in 3 per cent. of the cases. Dr. Lapage regards children so affected as instances of mild Mongolism. This conclusion is supported by the fact that such obliquity is a feature of a bright type of amentia, and it is well recognised that Mongolian idiots are generally of the bright and merry order. Prognathism, on the other hand, which appears with about equal frequency, is allied to a dull and heavy imbecility.

Defects of balance and carriage are common among children of feeble intellect; a rolling gait was noticeable in a considerable number, although physical exercises and drill had done much to improve the majority.

Dr. Lapage concludes that feeble-minded children exhibit physical defects far more often than do children of average development, and that these defects are in such subjects commonly double or triple; that detailed and exact measurements of the head will often bring out unsuspected asymmetry and deficiencies of the skull, and that feeble-minded children are in general smaller and more poorly developed than ordinary school children, the disparity increasing as age advances.

[1] Med. Chron. Aug. 1905.

